# Supplementary material for: Coexisting divergent and convergent plate boundary assemblages indicate plate tectonics in the Neoarchean
Source: Nat Commun. 2022 Oct 28;13:6450. doi: 10.1038/s41467-022-34214-8 (PMC9616927; doi:10.1038/s41467-022-34214-8)
Supplement: Supplementary file 1 — Supplementary Information [file 41467_2022_34214_MOESM1_ESM.pdf]

Supplementary Information for

Coexisting divergent and convergent plate boundary assemblages indicate plate tectonics in the Neoarchean

Bo Huang*, Tim E. Johnson, Simon A. Wilde, Ali Polat, Dong Fu, Timothy Kusky*

*Corresponding author. Email: hbyjdg@cug.edu.cn (B.H.); tkusky@gmail.com (T.K.)

The supplementary information includes:

Supplementary Note 1: Geological background of the North China Craton and Dengfeng-Angou complexes

Supplementary Note 2: Evaluation of element mobility

Supplementary Note 3: Zircon U–Pb geochronology and Lu–Hf isotope

Supplementary Note 4: Estimation of mantle potential temperatures (T_p)

Supplementary Figures: Figs. S1 to S14

Supplementary references

Supplementary Note

1. Geological background of the North China Craton and Dengfeng-Angou complexes

The North China Craton (NCC) is one of the largest cratonic blocks and records a long-term evolutionary history tracking back to at least 3.8 Ga [1-3]. The Precambrian basement of the NCC is generally subdivided into the Eastern and Western blocks, and the intervening orogenic belt, which is termed the Neoarchean Central Orogenic Belt[4,5] or Paleoproterozoic Trans-North China Orogen[6,7], along with several Paleoproterozoic tectonic belts (Fig. S2a in Supplementary Infomation). The basement rocks in the eastern NCC are dominated by Neoarchean (ca. 2.85–2.5 Ga) plutonic rocks of diverse origins and subordinate volcanic and sedimentary rocks, with local Eoarchean to Mesoarchean (ca. 3.8–2.9 Ga) rocks[3,6,8-10]. The eastern NCC (Eastern Block) was largely formed by accretion of several microcontinental nuclei, oceanic plateau and/or arc terranes during the Neoarchean (2.7–2.5 Ga)[4,5,9-11]. The western NCC (Western Block) is covered by thick Mesozoic sedimentary rocks (the Ordos Basin), and the exposed basement along its northern and northwestern margins is dominated by Neoarchean and Paleoproterozoic rocks[4].

The Central Orogenic Belt mainly comprises Neoarchean (dominantly ca. 2.56–2.50 Ga, with minor 2.7–2.6 Ga) TTG gneisses, Neoarchean to Paleoproterozoic metavolcano-sedimentary rocks and granitoids[4]. The majority of Neoarchean magmatic rock associations in the Central Orogenic Belt (Fig. S2a) include TTG gneisses, tholeiitic and calc-alkaline volcanic and plutonic rocks, and high-Mg diorite, which have been widely regarded as subduction-related arc rocks[11-15]. Several Neoarchean ophiolitic/tectonic mélanges and podiform chromitites with a forearc origin have been documented along the Central Orogenic Belt, marking a Neoarchean suture zone between a Central arc system and the Eastern Block[14-20]. Intense 2.52–2.48 Ga metamorphism[21-24], 2.5–2.45 Ga K-rich granitoids[25], and unconformably-overlying Paleoproterozoic sedimentary rocks provide further evidence for the amalgamation and cratonization of the Proto-North China Craton (at least including the central and eastern NCC) in the late Archean to early Paleoproterozoic (2.52–2.45 Ga)[26].

The Dengfeng and Angou complexes (Fig. S2b) in the southern segment of the Central Orogenic Belt have been regarded as a classic granite–greenstone belt, with a well-exposed lithostructural section and weak overprinting by Paleoproterozoic metamorphism, thus providing an excellent window for exploring Archean geodynamic processes[12,13,26-28]. In early studies, the granite–greenstone belts were generally interpreted as an Archean to Paleoproterozoic intraplate rock association formed either in a continental rift or dome-and-keel-like setting[27,28]. However, recent high-precision geochronological and geochemical data have shown that the Dengfeng and Angou complexes mainly formed at 2.55–2.50 Ga and are considered to be subduction-related[12,13,29,30]. More recently, integrating detailed field mapping with geochemical and metamorphic investigations, Huang et al.[21,26] recognized two lithostructural units that have distinct lithological, structural, metamorphic, and geochemical characteristics in the Dengfeng Complex, including: (1) an upper plate intra-oceanic arc/forearc complex consisting of subduction initiation-related forearc metagabbro–metabasalt, and island-arc-affinity TTG gneiss, high-Mg diorite

(sanukitoid) and adakitic sill/dikes, with high temperature/pressure (*T/P*) anatexis; and (2) a forearc accretionary complex derived from the lower plate consisting of structurally-repeated metabasalt–chert–shale ocean plate stratigraphy, trench-fill turbidites, and block-in-matrix mélanges, with intermediate *T/P* metamorphism recorded in entrained garnet amphibolite and metapelite blocks/sheets. The high *T/P* anatexis in the arc/forearc region, together with the intermediate *T/P* metamorphism in the accretionary complex, indicate a Neoarchean paired metamorphic belt[26]. The documentation of accretionary complex and paired metamorphism in the Dengfeng Complex provides positive evidence for asymmetric subduction processes in the late Archean.

Nevertheless, the linked system of plate subduction and seafloor spreading that is the hallmark feature of horizontal plate tectonics has not been previously documented, largely due to a lack of recognition of temporally- and spatially-related lithostructural associations formed in the supra-subduction zone (SSZ) and mid-ocean ridge (MOR)–passive margin. In this contribution, we document a new profile from the Angou Complex that contains lithostructural assemblages with MOR–passive margin and SSZ origins, reflecting seafloor spreading and plate convergence, respectively, and providing unequivocal direct evidence for the divergent and convergent plate-boundary processes and thus the operation of plate tectonics in the late Archean.

2. Evaluation of element mobility

The Angou Complex has undergone greenschist to lower amphibolite facies metamorphism, and thus it is necessary to evaluate potential element mobility before undertaking petrogenetic and geodynamic interpretations. First, samples with a high loss on ignition (LOI, >5 wt%, Supplementary Data 1) and minor volcanic rock samples containing amygdaloidal calcite were dismissed. Most other samples have relatively uniform normalized multi-elemental distribution patterns (Fig. 2c–h), and both high field strength elements (HFSEs, e.g., Nb, Ta, Zr, Hf and Ti) and rare earth elements (REEs) show good correlations with the immobile elements Nb and Zr (not shown). It is well documented that these immobile elements (e.g., HFSEs and REEs) are difficult to exchange during metamorphism and alteration[31,32]. In the chondrite-normalized REE diagrams, different types of samples all show relatively consistent REE patterns (Fig. 2c, e, g), and the Ce/Ce* values of most samples are between 0.9 and 1.1, suggesting that REEs have not undergone significant modification[31]. Such immobile elements have been used as the key geochemical proxy in the interpretation.

In contrast, some major elements (e.g., Na_2O, K_2O) and large ion lithophile elements (LILEs) show stronger mobility during post-magmatic alteration and metamorphism[31]. These elements in different groups of samples are relatively scattered in the element variation diagrams, suggesting that they have been modified to a certain extent, and so are generally not used in the discussion of petrogenesis. However, some LILEs (e.g., Ba) in mafic rocks may not have been significantly modified by post-magmatic processes, based the on following lines of evidence: 1) Ba and Ba/Th in metabasaltic rocks show good correlations with immobile elements like Nb (not shown); and 2) they plot along linear

trends or cluster in concentrated fields (Fig. 3c). If these elements or elemental ratios had been significantly modified by post-magmatic regional metamorphism, then uniform geochemical characteristics would be expected. Therefore, we consider the Ba/Th ratio of metabasaltic rocks, coupled with other immobile element ratios, to be a useful and additional proxy in fingerprinting the mantle source characteristics of these mafic rocks.

Collectively, HFSEs, REEs, and some LILEs in mafic rocks are used to characterize the petrogenesis and provenance characteristics of rocks in the Angou Complex.

3. Zircon U–Pb geochronology and Lu–Hf isotope

Twelve samples were selected for zircon U–Pb dating and Lu–Hf isotope analysis (see Methods and Supplementary Data 2–3). The sample locations and field photographs are shown in Fig. 1b and Fig. S9, respectively. The results of zircon U–Pb isotopic dating and Lu–Hf isotopic analysis are described below. The age error is calculated at 2σ.

Sample 21AG02-2 was collected from a ~20-cm-thick felsic volcanic interlayer in thick sedimentary sequences of the Eastern Belt of the Angou Complex (Fig. S9a). Zircon grains are prismatic to stubby, with lengths of ~80–100 μm and length/width ratios from 2:1 to 3:1. They exhibit good oscillatory zoning in cathodoluminescence (CL) images (Fig. S10). All analyses have moderate to high concentrations of Th (15–309 ppm) and U (45–272 ppm), with Th/U ratios from 0.32 to 1.82. Twenty-two analyses plot along a discordia line and yield an upper intercept age of 2530 ± 16 Ma (2σ, MSWD=0.49), similar, within error, to the weighted mean $^{207}Pb/^{206}Pb$ age of 2523 ± 13 Ma calculated using nineteen near-concordant analyses (Fig. S11a). Ten analyses of Lu–Hf isotopes yield positive $\varepsilon_{Hf(t)}$ values (+4.2 to +6.4).

Sample 22AG04 was collected from a ~80-cm-wide dacite porphyry that is parallel to the metabasalt of the Eastern Belt of the Angou Complex (Fig. S9b). Most zircon grains are prismatic, with length/width ratios of 2:1–3:1, and they exhibit oscillatory zoning (Fig. S10). The analyzed zircons have Th contents of 8–79 ppm and U contents of 28–98 ppm and Th/U ratios of 0.28–0.91. Seventeen analyses yield a weighted mean $^{207}Pb/^{206}Pb$ age of 2527 ± 17 Ma (MSWD=0.36) (Fig. S11b). Ten analyses of Lu–Hf isotopes yield positive $\varepsilon_{Hf(t)}$ values (+3.4 to +4.9).

Sample 22AG08 was collected from a granite dike that intruded the Eastern Belt of the Angou Complex (Fig. S9c). Zircon grains are prismatic, with lengths of ~80–100 μm and length/width ratios from 1:1 to 3:1. They exhibit consistent oscillatory zoning (Fig. S10). All analyses have Th contents of 13–109 ppm and U contents of 28–138 ppm, with Th/U ratios from 0.25 to 0.91. Twenty-three analyses plot along a discordia line and yield an upper intercept age of 2504 ± 16 Ma (MSWD=0.1), similar, within error, to the weighted mean $^{207}Pb/^{206}Pb$ age of 2504 ± 15 Ma calculated using twenty-one near-concordant analyses (Fig. S11c). Ten analyses of Lu–Hf isotopes yield positive $\varepsilon_{Hf(t)}$ values (+3.2 to +4.2).

Metadacite samples (18AG05-3, 18AG03-1, 18AG06-4) were collected from the volcanic

succession of the Central Belt of the Angou Complex (Fig. S9d–f). Zircon grains from these samples have similar morphology and structure, being dominantly prismatic to stubby, with length/width ratios from 1:1 to 3:1. Most zircons exhibit oscillatory zoning. They have variable contents of Th (36–145 ppm, 38–119 ppm and 34–87 ppm), U (59–323 ppm, 55–110 ppm and 54–93 ppm) and high Th/U ratios (0.32–1.05, 0.62–1.07 and 0.57–0.94), respectively, consistent with a magmatic origin. Fifteen analyses on sample 18AG05-3 plot along a discordia line and yield an upper intercept age of 2537 ± 19 Ma (MSWD=0.12), within error of the weighted mean $^{207}Pb/^{206}Pb$ age of 2525 ± 25 Ma, calculated using eight near-concordant analyses (Fig. S11d). Six analyses of Lu–Hf isotopes yield positive $\varepsilon_{Hf(t)}$ values (+6.1 to +7.4). Fifteen analyses on sample 18AG03-1 plot along a discordia line and yield an upper intercept age of 2529 ± 24 Ma (MSWD=0.07), which is within error of the weighted mean $^{207}Pb/^{206}Pb$ age of 2522 ± 23 Ma calculated using twelve near-concordant analyses (Fig. S11e). Six analyses of Lu–Hf isotopes yield positive $\varepsilon_{Hf(t)}$ values (+4.0 to +6.3). Fifteen analyses on sample 18AG06-4 record an upper intercept age of 2525 ± 23 Ma (MSWD=0.13), within error of the weighted mean $^{207}Pb/^{206}Pb$ age of 2515 ± 19 Ma (Fig. S11f). Eight analyses of Lu–Hf isotopes yield positive $\varepsilon_{Hf(t)}$ values (+4.9 to +6.2).

A quartz-rich schist sample 18RZ03-1 was collected from a metapelite interlayer of metabasaltic rocks in the western part of the Central Belt of the Angou Complex (Fig. S9g). Zircon grains from this sample are stubby to prismatic. Most zircons exhibit broad or oscillatory zoning, and minor grains have core-rim structures, with structureless or oscillatory-zoning cores surrounded by brighter rims. The analyzed zircons have variable contents of Th (12–336 ppm), U (35–352 ppm), and Th/U ratios (0.36–2.24). Sixteen analyses yield a weighted mean $^{207}Pb/^{206}Pb$ age of 2533 ± 25 Ma (MSWD=0.03) (Fig. S11g). Eight analyses of Lu–Hf isotope yield positive $\varepsilon_{Hf(t)}$ values (+4.5 to +7.3).

A K-rich granite dike sample 18AG08-4 was collected from the Central belt of the Angou Complex, intrusive into the volcano-sedimentary sequence (Fig. S9h). Zircons from this sample are mainly prismatic, with oscillatory zoning, and their length/width ratios range from 1.5:1 to 3:1. They have relatively high Th (114–480 ppm) and U (141–1116 ppm) contents, with Th/U ratios of 0.21–1.37. Ten analyses define a discordia line with an upper intercept age of 2499 ± 36 Ma (MSWD=0.06) (Fig. S11h), which is interpreted as the crystallization age of the dike. Six analyses of Lu–Hf isotopes yield positive $\varepsilon_{Hf(t)}$ values (+4.1 to +5.7).

Sample 22RZ06b was collected from an amphibolite lens in the Western Belt of the Angou Complex (Fig. S9i). The amphibolite is subparallel to the trondhjemitic gneisses (Fig. S7a). Zircons from this sample exhibit broad and sector zoning, and some have core-rim structures (Fig. S10). The analyzed zircons have variable contents of Th (0.4–27 ppm), U (10–77 ppm), and Th/U ratios (0.02–0.4). Twenty-three near-concordant analyses on magmatic zircons yield a weighted mean $^{207}Pb/^{206}Pb$ age of 2549 ± 18 Ma (MSWD=0.1) (Fig. S11i). Six near-concordant analyses on metamorphic zircons plot along a discordia line and yield a weighted mean $^{207}Pb/^{206}Pb$ age of 2483 ± 28 Ma (MSWD=0.1). Nine analyses of Lu–Hf isotopes on magmatic zircons yield nearly consistent positive $\varepsilon_{Hf(t)}$ values (+5.5 to +6.7).

Trondhjemitic gneiss sample 18RZ14-2 was collected from the Western Belt of the Angou Complex (Fig. 9j). Zircon grains are prismatic and relatively large, with lengths of ~100–200 μm and length/width ratios from 2:1 to 3:1. They exhibit good oscillatory zoning in CL images (Fig. S10). All analyses have moderate to high concentrations of Th (52–433 ppm) and U (88–451 ppm), with Th/U ratios from 0.33 to 0.96, suggesting they are of magmatic origin. Twelve analyses plot along a discordia line and yield an upper intercept age of 2538 ± 20 Ma (MSWD=0.06), similar, within error, to the weighted mean $^{207}Pb/^{206}Pb$ age of 2527 ± 26 Ma calculated using eight near-concordant analyses (Fig. S11j). The 2538 ± 20 Ma age is interpreted as the crystallization age of the trondhjemitic protolith. Six analyses of Lu–Hf isotopes yield positive $\varepsilon_{Hf(t)}$ values (+5.8 to +7.6).

TTG gneiss sample18RZ01-1 was also collected from the Western Belt of the Angou Complex (Fig. 9k). Zircon grains are elongated to stubby, with lengths of ~70–120 μm and length/width ratios from 1.2:1 to 2:1. Most zircons exhibit oscillatory zoning. All analyses have moderate concentrations of Th (39–143 ppm) and U (101–232 ppm), with Th/U ratios from 0.37 to 0.85. Fourteen analyses plot along a discordia line and yield an upper intercept age of 2544 ± 29 Ma (MSWD=0.07), similar, within error, to the weighted mean $^{207}Pb/^{206}Pb$ age of 2532 ± 24 Ma calculated on eight near-concordant analyses (Fig. S11k). Six analyses of Lu–Hf isotopes yield positive $\varepsilon_{Hf(t)}$ values (+5.7 to +6.9).

A leucogranitic vein sample 18RZ01-2 is associated with the TTG gneiss 18RZ01-1 (Fig. 9l). Zircons from this sample are prismatic or stubby, with oscillatory zoning. Twelve analyses yield variable Th and U contents and moderate to high Th/U ratios (0.42–1.88). They define a discordia line, with an upper intercept age of 2500 ± 24 Ma (MSWD=0.14) (Fig. S11l). This age is interpreted as the age of anatexis of the TTG protolith, and is consistent with the timing of regional metamorphism and anatexis in the Angou and Dengfeng complexes (Fig. S13). Six analyses of Lu–Hf isotope yield positive $\varepsilon_{Hf(t)}$ values (+5.4 to +6.7).

4. Estimation of mantle potential temperatures (T_p)

We estimated mantle potential temperature (T_p) using the major element compositions of basalts in the Angou Complex following the method of ref.[33]. The reconstructed primary mantle melts with compositions that were (mostly) affected by olivine fractionation[33] (Supplementary Data 9). The samples with LOI > 4 wt% are first excluded. Samples with MgO >7 wt% (except for several samples from island arc/forearc basalts with MgO >6.5%) and SiO_2 of 45–55 wt% were used to minimize the effects of pyroxene fractionation[34]. In the calculations, the addition of olivine to obtain the primary melt composition stops when a forsterite (Fo) content of 90 is reached. The calculations assume a bulk Fe^{3+}/Fe^T of 0.1, which corresponds to relatively reduced conditions during the Archean. To aid comparison, we assume an anhydrous system, which yields temperatures ~55–80 °C higher than for one calculated with 3 wt% H_2O (Supplementary Data 9, Fig. S14).

The MORB-like basalts from the Eastern Belt record T_p of ~1410–1500 °C (average ~1450 °C), up to ~100 °C higher than that of average modern MORB (~1350 °C; Fig. S14). Forearc basalts from the

Western Belt and high-Mg basalts from the Central Belt yield higher T_p of ~1465–1485 °C (average ~1475 °C) and 1400–1460 °C (average ~1430 °C), respectively, which may reflect heat associated with upwelling mantle beneath the forearc following subduction initiation[35]. In contrast, IAB-type rocks from the Central Belt have lower T_p of ~1310–1390 °C (average ~1340 °C), consistent with T_p of arc basalts from the Izu–Bonin arc/forearc (anhydrous, data from GEOROC) (Fig. S14). Similar T_p conditions have been documented for basalt–boninite sequences from the Izu–Bonin forearc, which yielded higher T_p (~1400–1480 °C, anhydrous, Fe^{3+}/Fe^T = 0.15, Fo = 90) for forearc basalts[35] and lower T_p (~1340 ± 60 °C, anhydrous, Fe^{3+}/Fe^T = 0.21, Fo = 91) for younger boninitic rocks[36].

Supplementary Figures

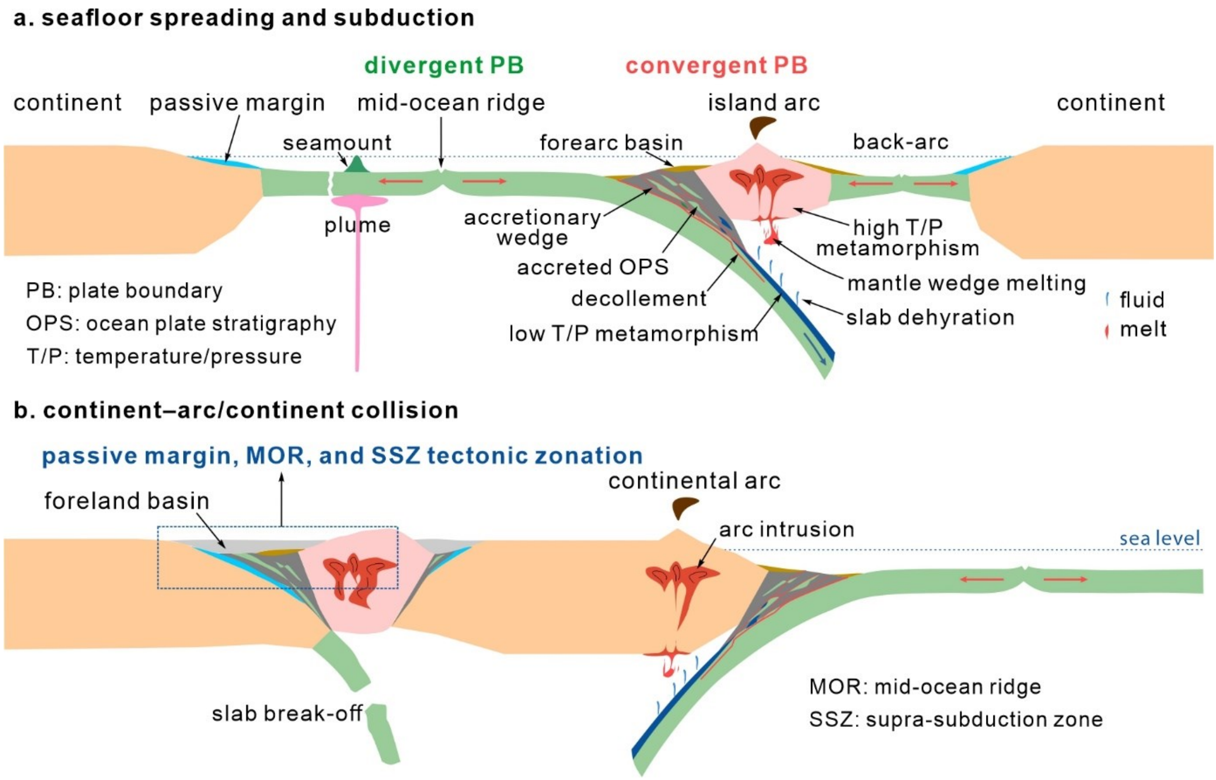

Fig. S1 Two-dimension cartoons showing ideal divergent (seafloor spreading) and convergent (subduction and collision) processes within the modern plate tectonic framework. (**a**) Passive margin sedimentation and mid-ocean ridge and back-arc spreading mark the extensional process, whereas the self-sustaining subduction of oceanic lithosphere and generation of arcs represent the convergent process. Ocean plate stratigraphy (basalt–chert–shale ± limestone) and exhumed low *T/P* metamorphic rocks would be accreted onto the accretionary wedge (if it is an accretionary margin). The low *T/P* metamorphism along the subduction zone and high *T/P* metamorphism at the arc/back-arc would constitute paired metamorphic belts, signifying asymmetric subduction. Slab fluid-fluxed partial melting of the mantle wedge results in arc magmatism. Slab melting would occur in some rare instances where there is subduction of hot and young crust (<20 Myr). (**b**) Continuous subduction leads to collision between continents and/or arcs, forming collisional orogenic belts that are characterised by diagnostic tectonic zonation across the orogen. The foreland basin sequence then overlaps the assembled orogenic wedge and craton margin. An intra-oceanic island arc and a continental arc are marked in panels a and b, respectively.

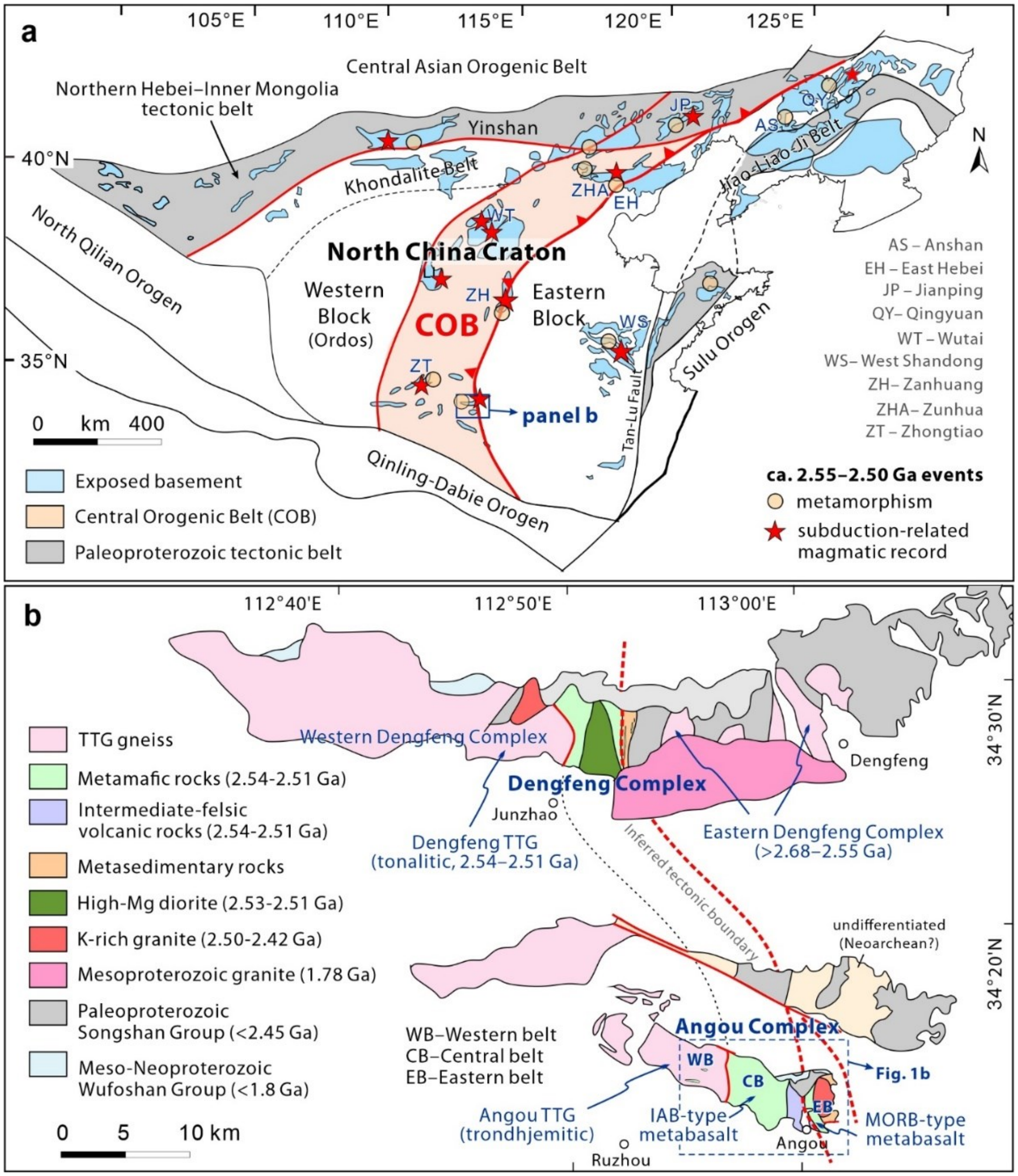

Fig. S2 (**a**) Tectonic subdivision of the North China Craton showing 2.55–2.50 Ga metamorphic and subduction-related magmatic events (modified from refs.[4,26]). (**b**) Simplified geological map showing the Dengfeng and Angou complexes (modified from refs.[21,30]). The 2.55–2.51-Ga Angou Complex is coeval with the Western Dengfeng Complex (2.55–2.51 Ga)[21].

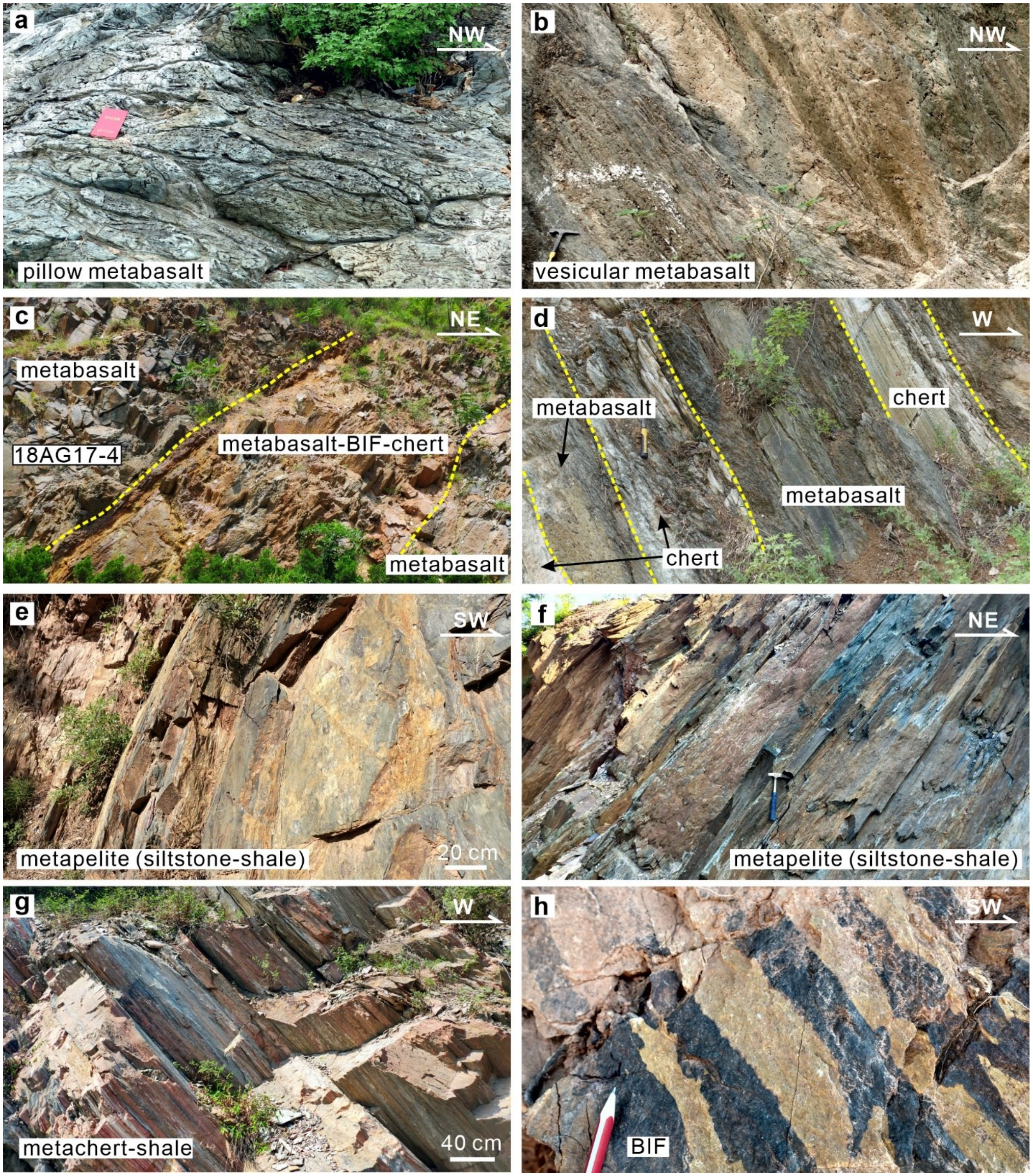

Fig. S3 Field photographs showing the ocean plate stratigraphy (**a–d**) and the passive margin sequence (**e–h**) in the Eastern Belt of the Angou Complex. (**a**) Relict pillow metabasalt; (**b**) vesicular metabasalt; (**c**) massive metabasalt–chert–shale–banded iron formation (BIF); (**d**) vesicular metabasalt–chert; (**e–f**) metapelite; (**g**) metachert–shale (quartz-mica schist, quartz schist); and (**h**) BIF interbedded with quartz-rich schists.

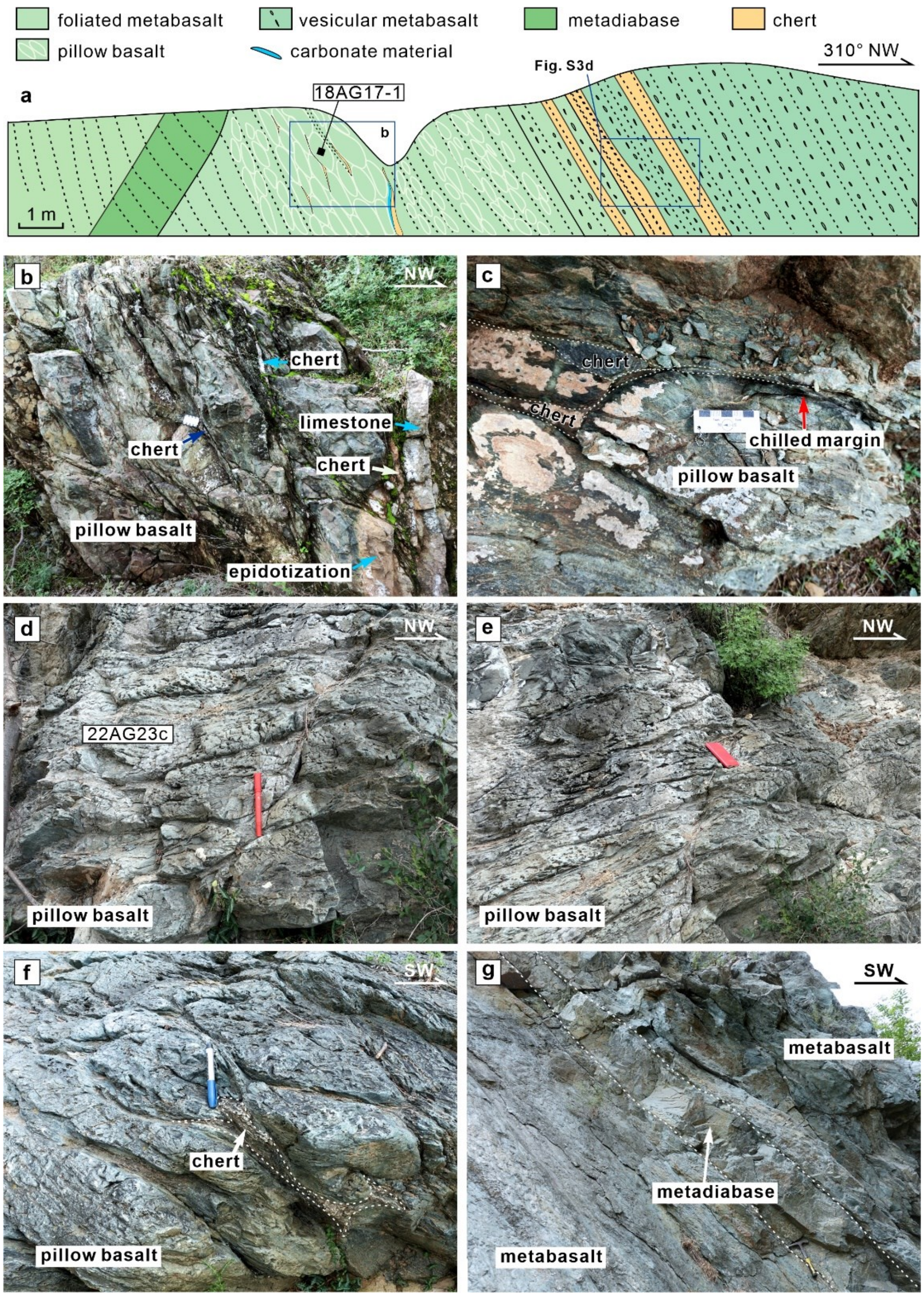

Fig. S4 A simplified lithostructural cross-section (**a**) and field photographs showing outcrops of relict pillow metabasalts, with chert and/or carbonate filling the chilled margins (selvedge) of pillows (**b–f**), and a metadiabase intruding the metabasalt (**g**) in the Eastern Belt of the Angou Complex.

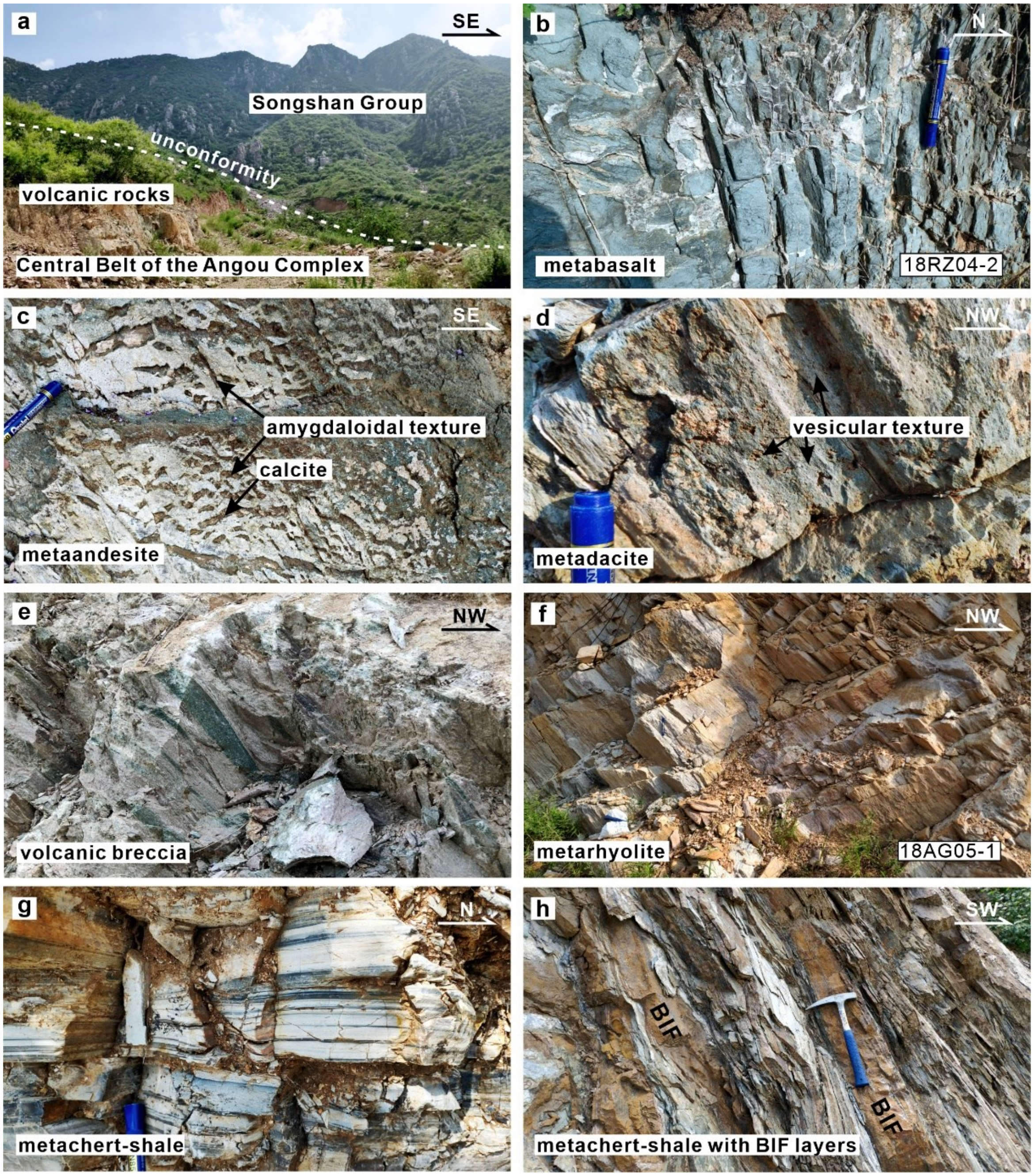

Fig. S5 Field photographs showing metavolcano-sedimentary rocks in the Central Belt of the Angou Complex. (**a**) The unconformity between the Songshan Group and the Central Belt of the Angou Complex, (**b**) foliated metabasalt (amphibolite), (**c**) metaandesite with amygdaloidal textures, (**d**) metadacite with vesicular texture, (**e**) deformed volcanic breccia, (**f**) metarhyolite, and (**g–h**) well-bedded metachert-shale-BIF sequences.

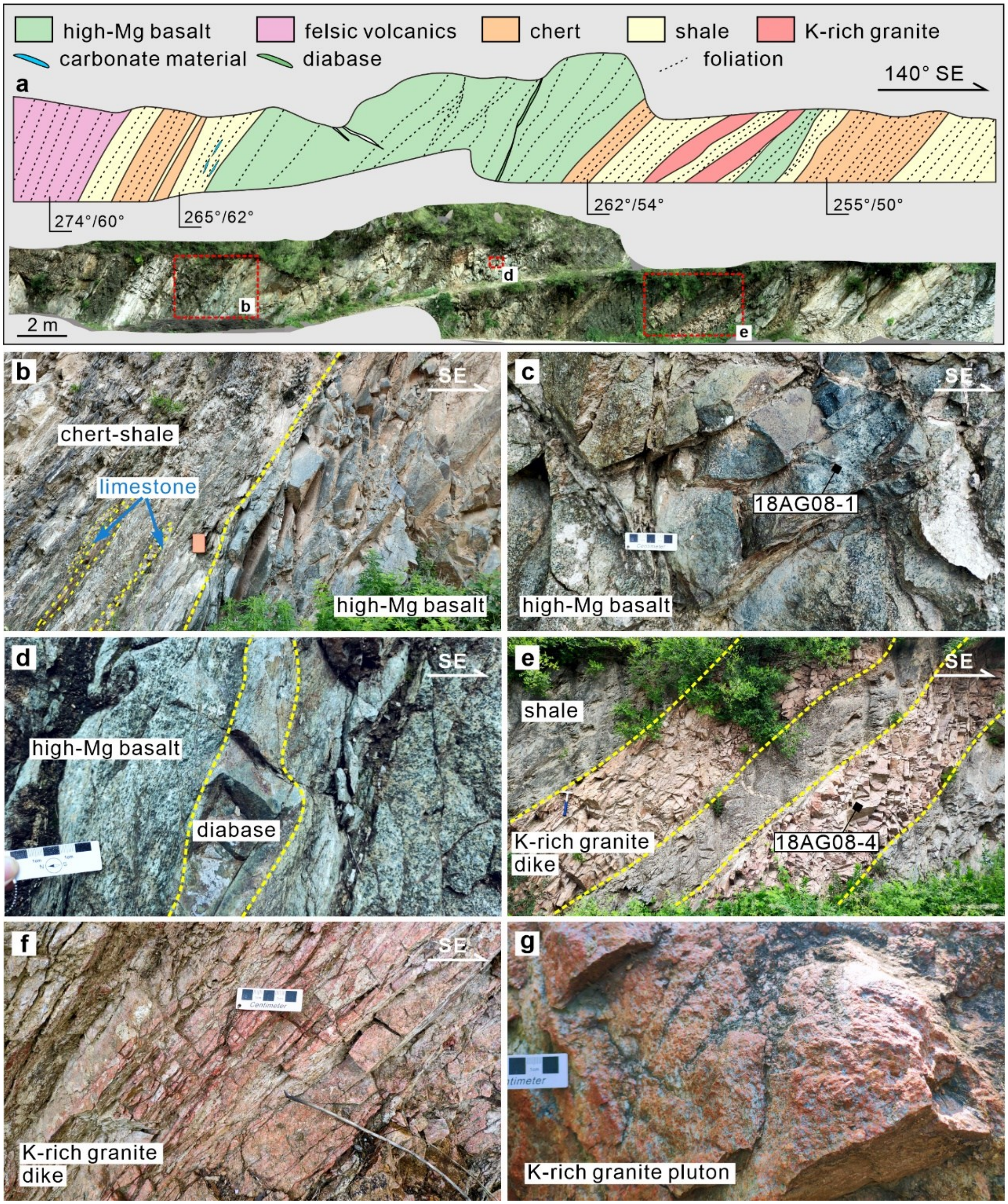

Fig. S6 Lithostructural section (**a**) and field photographs (**b–g**) of the Central and Eastern belts of the Angou Complex. (**a**) Lithostructural section showing high-Mg basalt and associated volcano-sedimentary sequences in the Central Belt. (**b–d**) High-Mg metabasalt, diabase, chert, and shale in the Central Belt. (**e–f**) Post-kinematic K-rich granite dikes in the Central Belt. (**g**) Circa 2.51–2.50 Ga K-rich granite pluton intruding the metabasalt–chert–shale sequence of the Eastern Belt.

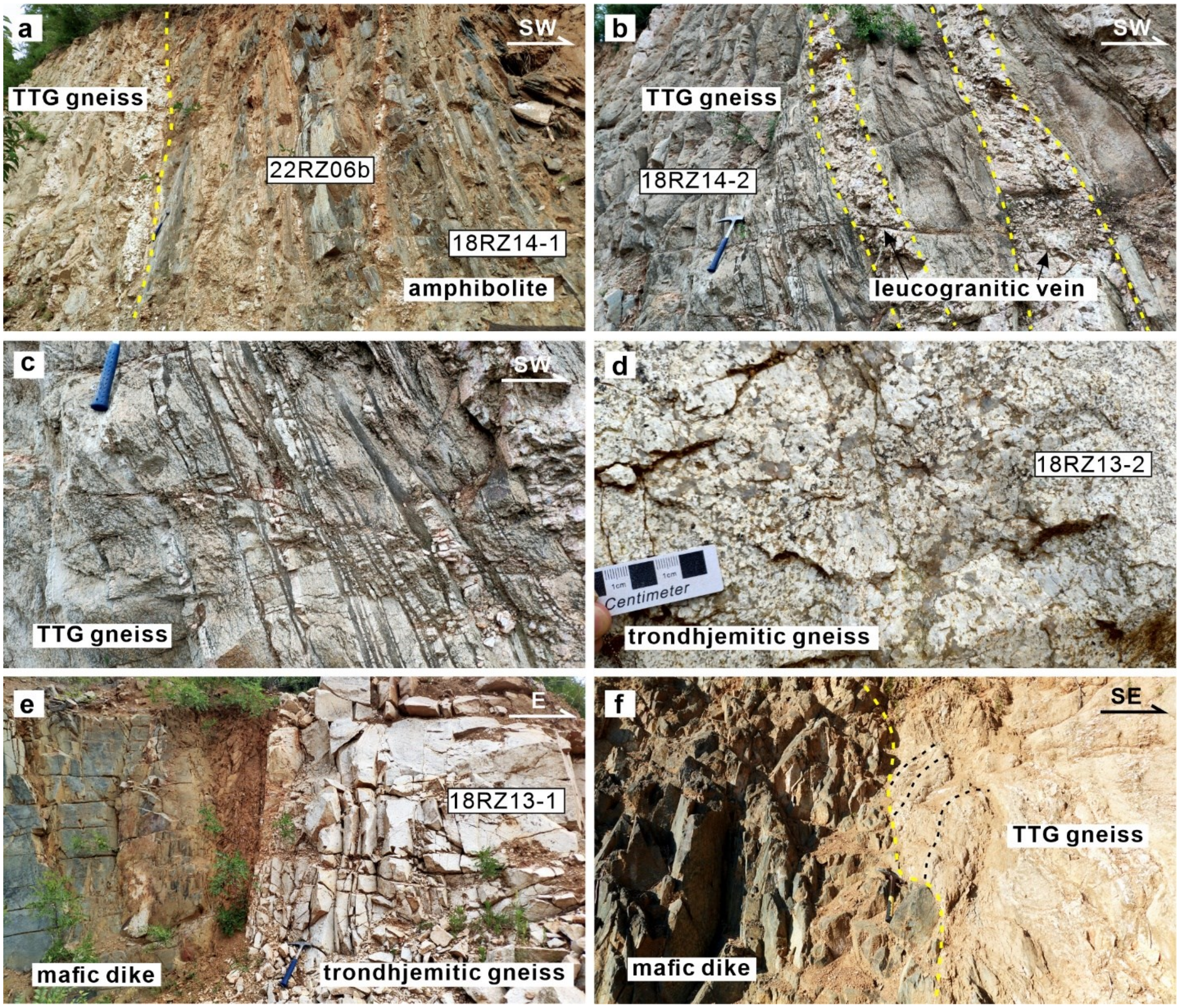

Fig. S7 Field photographs showing typical lithologies in the Western Belt of the Angou Complex. (**a–d**) TTG gneisses, metabasites (amphibolites) with leucogranitic veins. (**e–f**) Mafic dikes intruding the TTG gneisses.

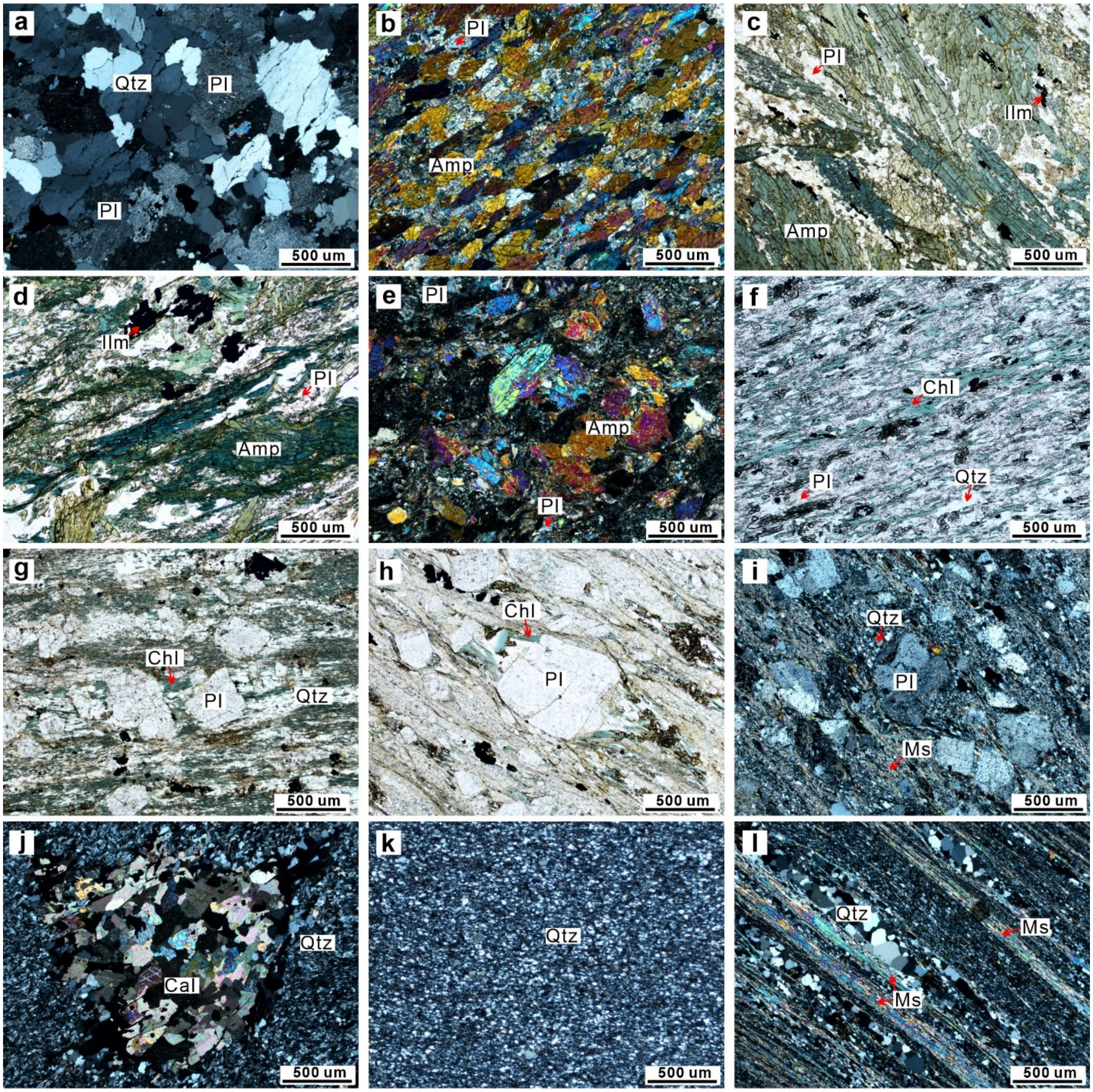

Fig. S8 Photomicrographs illustrating the diverse lithologies in the Angou Complex. (**a**) Trondhjemtitic gneiss (18RZ13-1) and (**b**) amphibolite boudin (18RZ14-1) in the Western Belt. (**c–d**) Metabasalts (18AG09-1) in the Eastern Belt. (**e**) High-Mg basalt (18AG08-1), (**f**) metaandesite (18AG17-6), and (**g–i**) metadacites (18AG05-2, 18AG20-2, and 18AG06-4, respectively) with sheared feldspars and (**j**) amygdaloidal structure, with calcite amygdale (18AG05-4) from the Central Belt. (**k**) Metachert and (**l**) quartz-mica schist (metasiltstone/shale, 18AG15-2) from the Eastern Belt. Mineral abbreviations: Qtz, quartz; Pl, plagioclase; Amp, amphibole; Ilm, ilmenite; Chl, chlorite; Ms, muscovite; and Cal, calcite.

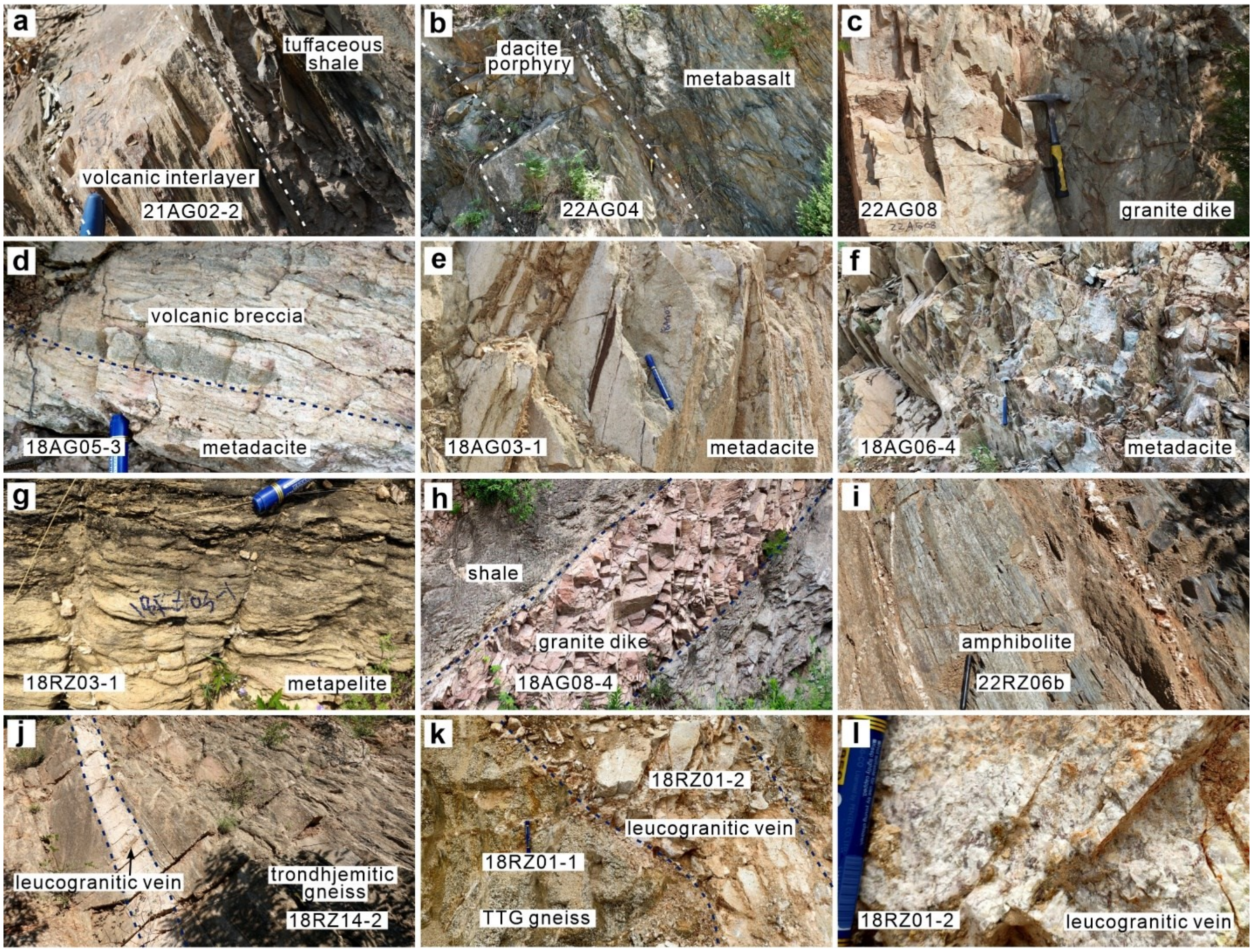

Fig. S9 Field photographs showing the geochronological samples from the Angou Complex.

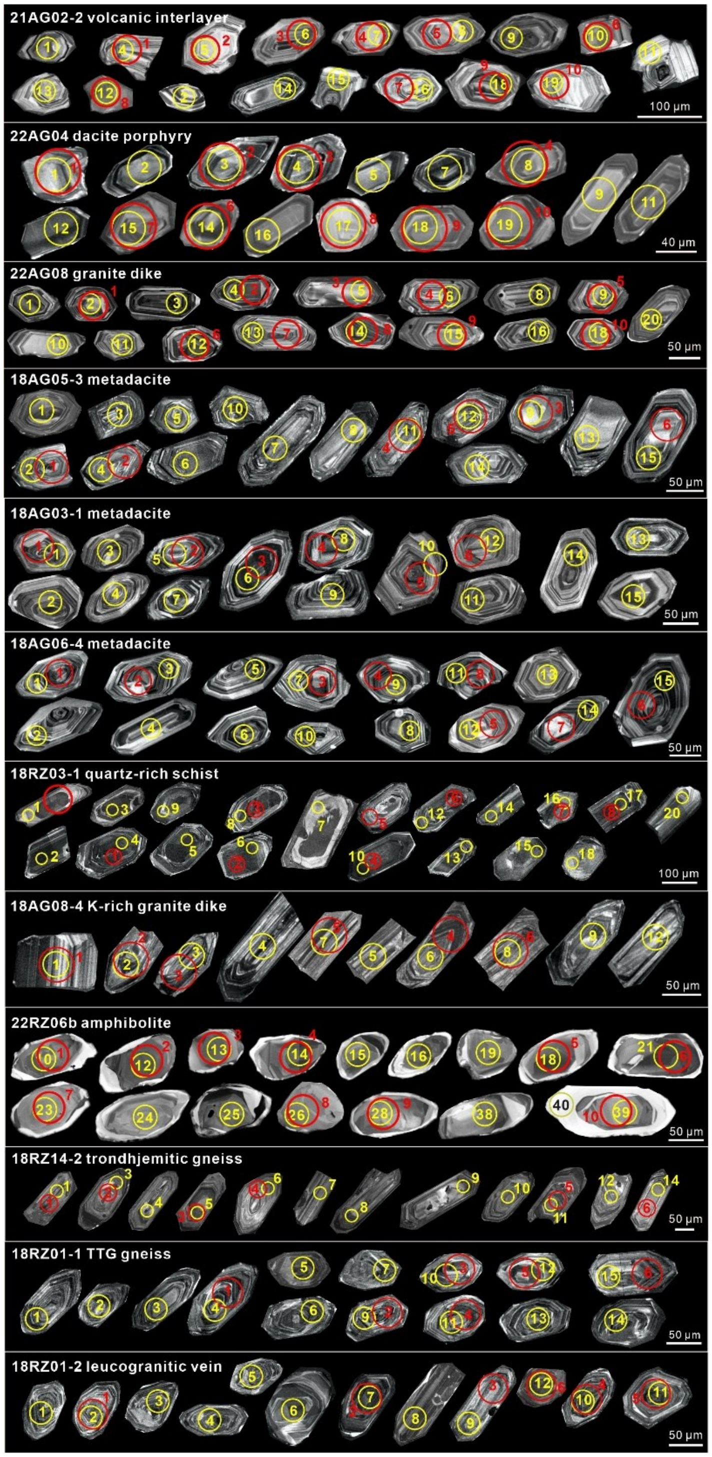

Fig. S10 Cathodoluminescence images of zircons in the analysed samples from the Angou Complex. Most zircon grains from trondhjemitic gneisses, metavolcanic rocks, and dikes exhibit well-developed oscillatory zoning, indicating a magmatic origin. Yellow and red circles represent the spot locations of U–Pb (diameter = 32 μm) and Lu–Hf analyses (diameter = 44 μm), respectively.

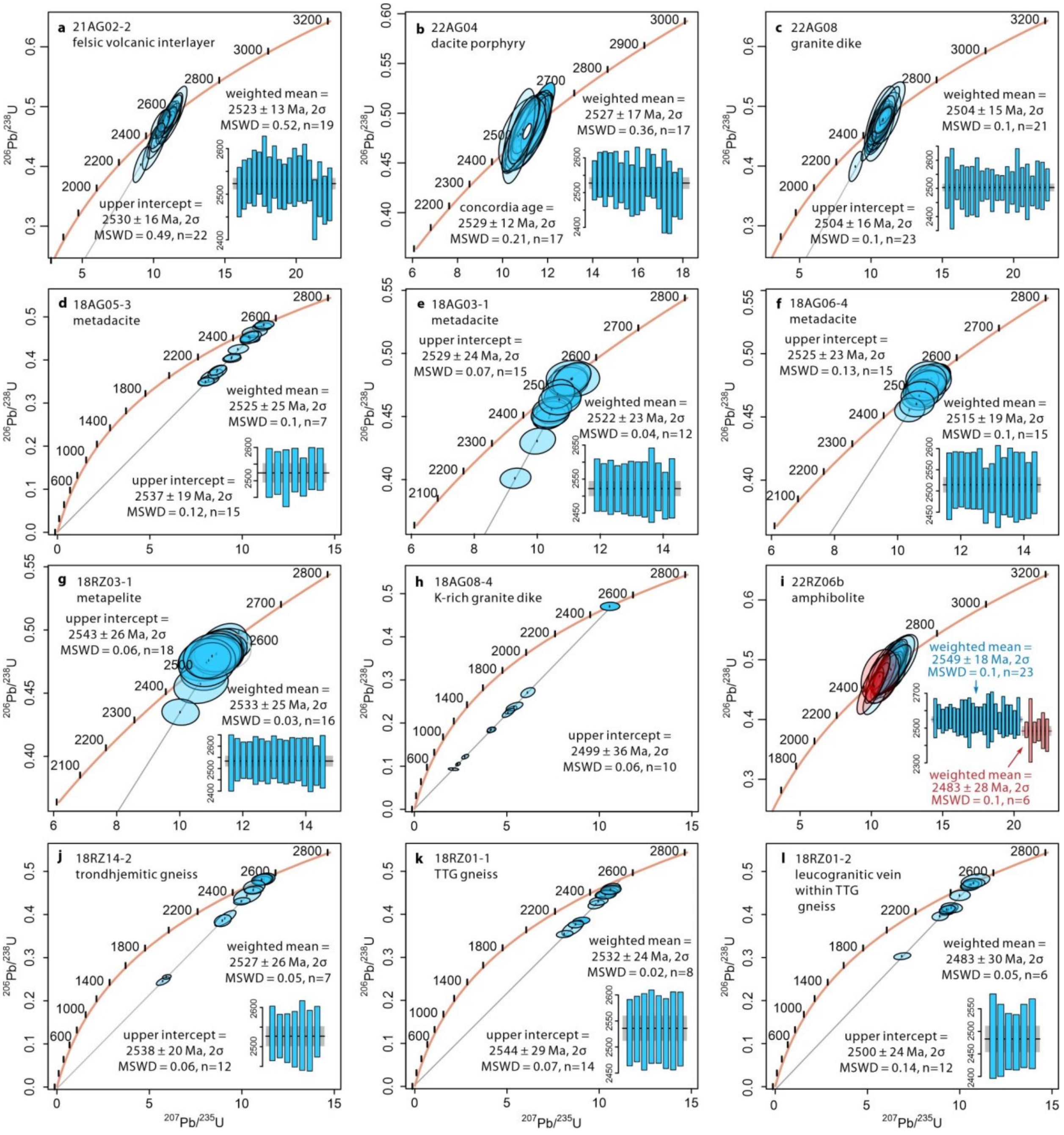

Fig. S11 Zircon U–Pb concordia diagrams, upper intercept ages, and weighted mean $^{207}Pb/^{206}Pb$ ages of samples from the Angou Complex. The uncertainty of data-point plots and calculated ages is 2σ. (**a**) Volcanic interlayer in the quartz-mica schist, and (**b**) dacite porphyry within metabasalt in the Eastern Belt. (**c**) Granite dike from the Eastern Belt. (**d–f**) Metadacites, (**g**) metapelite, and (**h**) K-rich granitic dike from the Central Belt. (**i**) Amphibolite sheet, (**j**) trondhjemitic gneiss, (**k**) TTG gneiss, and (**l**) leucogranitic vein in TTG gneiss in the Western Belt.

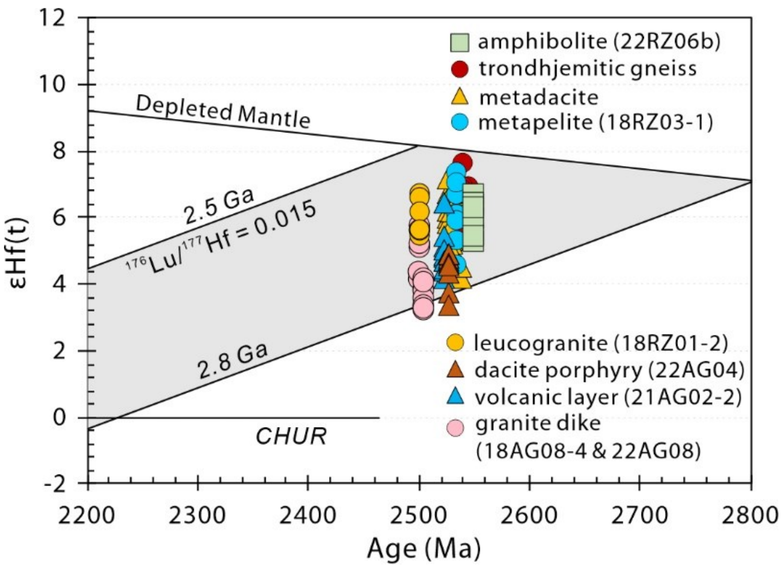

Fig. S12 Zircon $\varepsilon_{Hf}(t)$ versus age diagram for samples from the Angou Complex.

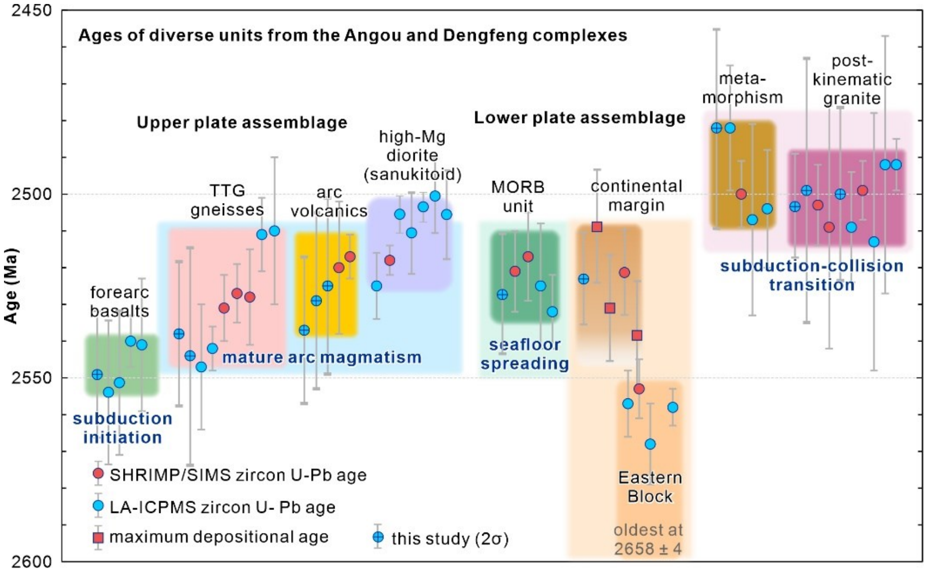

Fig. S13 Summary of ages of different lithological units in the Angou and Dengfeng complexes. Data and references of zircon ages are listed in Supplementary Data 8.

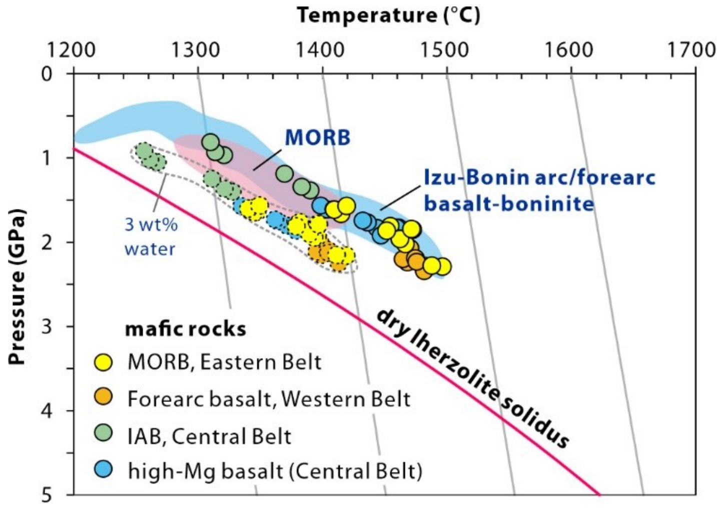

Fig. S14 Temperatures and pressures of generation of the mafic rocks in the Angou Complex, using the method of [33]. *P–T* arrays for anhydrous MORB and Izu-Bonin arc basalt–boninite (GEOROC) are shown for comparison, along with the dry peridotite solidus[37]. The addition of 3 wt% water (dotted circles) yielded lower temperatures than anhydrous conditions (solid circles).

Supplementary references

1. Liu, D. Y., Nutman, A. P., Compston, W., Wu, J. S. & Shen, Q. H. Remnants of ≥3800 Ma crust in the Chinese part of the Sino-Korean craton. *Geology* **20**, 339 (1992).
2. Zhai, M. Multi-stage crustal growth and cratonization of the North China Craton. *Geosci. Frontiers*. **5**, 457-469 (2014).
3. Zhao, G. et al. Amalgamation of the North China Craton: Key issues and discussion. *Precambrian Res.* **222-223**, 55-76 (2012).
4. Kusky, T. M. et al. Insights into the tectonic evolution of the North China Craton through comparative tectonic analysis: A record of outward growth of Precambrian continents. *Earth-Sci. Rev.* **162**, 387-432 (2016).
5. Kusky, T. M. & Li, J. Paleoproterozoic tectonic evolution of the North China Craton. *J. Asian Earth Sci.* **22**, 383-397 (2003).
6. Zhao, G., Sun, M., Wilde, S. A. & Li, S. Late Archean to Paleoproterozoic evolution of the North China Craton: key issues revisited. *Precambrian Res.* **136**, 177-202 (2005).
7. Zhao, G., Wilde, S. A., Cawood, P. A. & Sun, M. Archean blocks and their boundaries in the North China Craton: lithological, geochemical, structural and P–T path constraints and tectonic evolution. *Precambrian Res.* **107**, 45-73 (2001).

8. Wilde, S. A. The Precambrian Geology of the North China Craton: A Review and Update of the Key Issues. In: Dilek, Y. & Furnes, H., editors. *Modern Approaches in Solid Earth Sciences*. Dordrecht: Springer Science+Business Media; 2014. pp. 149-178.
9. Sun, G. et al. Thermal state and evolving geodynamic regimes of the Meso- to Neoarchean North China Craton. *Nat. Commun.* **12**, (2021).
10. Gao, L. et al. A Ca. 2.8-Ga Plume-Induced Intraoceanic Arc System in the Eastern North China Craton. *Tectonics* **38**, 1694-1717 (2019).
11. Wang, W. et al. Neoarchean intra-oceanic arc system in the Western Liaoning Province: Implications for Early Precambrian crustal evolution in the Eastern Block of the North China Craton. *Earth-Sci. Rev.* **150**, 329-364 (2015).
12. Deng, H. et al. A 2.5 Ga fore-arc subduction-accretion complex in the Dengfeng Granite-Greenstone Belt, Southern North China Craton. *Precambrian Res.* **275**, 241-264 (2016).
13. Diwu, C., Sun, Y., Guo, A., Wang, H. & Liu, X. Crustal growth in the North China Craton at ~2.5Ga: Evidence from in situ zircon U–Pb ages, Hf isotopes and whole-rock geochemistry of the Dengfeng complex. *Gondwana Res.* **20**, 149-170 (2011).
14. Polat, A. et al. Geochemical and petrological evidence for a suprasubduction zone origin of Neoarchean (ca. 2.5 Ga) peridotites, central orogenic belt, North China craton. *Geol. Soc. Am. Bull.* **118**, 771-784 (2006).
15. Polat, A. et al. Geochemistry of Neoarchean (ca. 2.55–2.50 Ga) volcanic and ophiolitic rocks in the Wutaishan greenstone belt, central orogenic belt, North China craton: Implications for geodynamic setting and continental growth. *Geol. Soc. Am. Bull.* **117**, 1387-1399 (2005).
16. Wang, J. et al. A late Archean tectonic mélange in the Central Orogenic Belt, North China Craton. *Tectonophysics* **608**, 929-946 (2013).
17. Kusky, T. et al. Mélanges through time: Life cycle of the world's largest Archean mélange compared with Mesozoic and Paleozoic subduction-accretion-collision mélanges. *Earth-Sci. Rev.* **209**, 103303 (2020).
18. Ning, W. et al. From subduction initiation to arc–polarity reversal: Life cycle of an Archean subduction zone from the Zunhua ophiolitic mélange, North China Craton. *Precambrian Res.* **350**, 105868 (2020).
19. Peng, H. et al. Identification of the Neoarchean Jianping pyroxenite-mélange in the Central Orogenic Belt, North China Craton: A fore-arc accretional assemblage. *Precambrian Res.* **336**, 105495 (2020).
20. Zhong, Y. et al. Alpine-style nappes thrust over ancient North China continental margin demonstrate large Archean horizontal plate motions. *Nat. Commun.* **12**, (2021).
21. Huang, B. et al. Structural relationships and kinematics of the Neoarchean Dengfeng forearc and accretionary complexes, southern North China craton. *Geol. Soc. Am. Bull.* **131**, 966-996 (2019).

22. Wang, C., Song, S., Allen, M. B., Su, L. & Wei, C. High-pressure granulite from Jixian, Eastern Hebei, the North China Craton: implications for Neoarchean to early Paleoproterozoic collision tectonics. *Geol. Soc. Spec. Publ*. **478**, 427-448 (2019).
23. Ning, W., Kusky, T., Wang, L. & Huang, B. Archean eclogite-facies oceanic crust indicates modern-style plate tectonics. *Proc. Natl Acad. Sci. USA* **119**, e2117529119 (2022).
24. Wu, Z. et al. Ultrahigh-pressure peridotites record Neoarchean collisional tectonics. *Earth Planet. Sc. Lett.* **596**, 117787 (2022).
25. Zhou, Y., Zhao, T., Wang, C. Y. & Hu, G. Geochronology and geochemistry of 2.5 to 2.4Ga granitic plutons from the southern margin of the North China Craton: Implications for a tectonic transition from arc to post-collisional setting. *Gondwana Res.* **20**, 171-183 (2011).
26. Huang, B. et al. Paired metamorphism in the Neoarchean: A record of accretionary-to-collisional orogenesis in the North China Craton. *Earth Planet. Sc. Lett.* **543**, 116355 (2020).
27. Kröner, A., Compston, W., Zhang, G. & Guo, A. Age and tectonic setting of Late Archean greenstone-gneiss terrain in Henan Province, China, as revealed by single-grain zircon dating. *Geology* **16**, 211-215 (1988).
28. Zhang, G. et al. Composition and evolution of the archaean crust in central Henan, China. *Precambrian Res.* **27**, 7-35 (1985).
29. Wang, X., Huang, X., Yang, F. & Luo, Z. Late Neoarchean magmatism and tectonic evolution recorded in the Dengfeng Complex in the southern segment of the Trans-North China Orogen. *Precambrian Res.* **302**, 180-197 (2017).
30. Zhang, J., Zhang, H., Li, L. & Wang, J. Neoarchean-Paleoproterozoic tectonic evolution of the southern margin of the North China Craton: Insights from geochemical and zircon U–Pb–Hf–O isotopic study of metavolcanic rocks in the Dengfeng complex. *Precambrian Res.* **318**, 103-121 (2018).
31. Polat, A. & Hofmann, A. W. Alteration and geochemical patterns in the 3.7–3.8 Ga Isua greenstone belt, West Greenland. *Precambrian Res.* **126**, 197-218 (2003).
32. Pearce, J. A. Geochemical fingerprinting of oceanic basalts with applications to ophiolite classification and the search for Archean oceanic crust. *Lithos* **100**, 14-48 (2008).
33. Lee, C. A., Luffi, P., Plank, T., Dalton, H. & Leeman, W. P. Constraints on the depths and temperatures of basaltic magma generation on Earth and other terrestrial planets using new thermobarometers for mafic magmas. *Earth Planet. Sc. Lett.* **279**, 20-33 (2009).
34. Condie, K. C., Aster, R. C. & van Hunen, J. A great thermal divergence in the mantle beginning 2.5 Ga: Geochemical constraints from greenstone basalts and komatiites. *Geosci. Frontiers*. **7**, 543-553 (2016).
35. Shervais, J. W. et al. Magmatic Response to Subduction Initiation: Part 1. Fore-arc Basalts of the Izu-Bonin Arc From IODP Expedition 352. *Geochem. Geophys. Geosyst.* **20**, 314-338 (2019).

36. Shervais, J. W. et al. Magmatic Response to Subduction Initiation, Part II: Boninites and Related Rocks of the Izu-Bonin Arc From IODP Expedition 352. *Geochem. Geophys. Geosyst.* **22**, e2020G-e9093G (2021).
37. Katz, R. F., Spiegelman, M. & Langmuir, C. H. A new parameterization of hydrous mantle melting. *Geochem. Geophys. Geosyst.* **4**, 1073 (2003).
