# Supplementary material for: Coexisting divergent and convergent plate boundary assemblages indicate plate tectonics in the Neoarchean
Source: Nat Commun. 2022 Oct 28;13:6450. doi: 10.1038/s41467-022-34214-8 (PMC9616927; doi:10.1038/s41467-022-34214-8)
Supplement: Supplementary file 3 — Description of Additional Supplementary Files [file 41467_2022_34214_MOESM3_ESM.docx]

Description of Additional Supplementary Files

**File name:** Supplementary Data 1

**Description:** Bulk-rock geochemical data of rocks in the Angou and Dengfeng complexes, southern North China Craton.

**File name:** Supplementary Data 2

**Description:** Zircon U-Pb isotopic data of the Angou Complex, southern North China Craton, and analysed standard zircons.

**File name:** Supplementary Data 3

**Description:** Zircon Lu-Hf isotopic data of the Angou Complex, southern North China Craton, and analysed standard zircons.

**File name:** Supplementary Data 4

**Description:** Bulk compositions used in thermodynamic and trace element modelling.

**File name:** Supplementary Data 5

**Description:** Mineral/melt partition coefficient used in trace element modelling.

**File name:** Supplementary Data 6

**Description:** Results of modal batch melting using the average compositions of MORB-type and IAB-type basaltic rocks from the Angou Complex, southern North China Craton.

**File name:** Supplementary Data 7

**Description:** Results of evolved melts after removal of two plausible fractionating mineral assemblages.

**File name:** Supplementary Data 8

**Description:** Summary of available geochronological data of the Angou and Dengfeng complexes in the southern North China Craton.

**File name:** Supplementary Data 9

**Description:** Temperatures and pressures of generation of the mafic rocks in the Angou Complex.
